# Synthetic Novel Flavonoids SZQ-4 Suppress Osteoclastogenesis and Ameliorate Osteoporosis via Inhibiting Reactive Oxygen Species and Regulating SIRT3

**DOI:** 10.3390/antiox15040426

**Published:** 2026-03-28

**Authors:** Runqi Zhou, Yichi Zhang, Bin Li, Mengjia Yi, Junhao Tu, Tianle Jiang, Haofu Jiang, Chaoming Hu, Yifan Ping, Jun Wang, Yixin Mao, Yang Chen, Zengqiang Song, Xian Tong, Shengbin Huang, Shufan Zhao

**Affiliations:** 1 Institute of Stomatology, School and Hospital of Stomatology, Wenzhou Medical University, Wenzhou 325027, China; zrq272727@163.com (R.Z.); zhangyichi0706@163.com (Y.Z.); binli0461@gmail.com (B.L.); myi2@ualberta.ca (M.Y.); tjhwmu@163.com (J.T.); jiangtl@wmu.edu.cn (T.J.); jianghaofu1999@163.com (H.J.); hcmwmu@163.com (C.H.); pingyifankouqiang@163.com (Y.P.); wj18325333305@163.com (J.W.); yx.mao@wmu.edu.cn (Y.M.); cylillie@163.com (Y.C.); tx847595271@163.com (X.T.); 2 Chemical Biology Research Center, School of Pharmaceutical Sciences, Wenzhou Medical University, Wenzhou 325035, China; songzengqiang09@163.com

**Keywords:** osteoclast differentiation, SIRT3 expression, osteoporosis, small-molecule flavonoid drug, SZQ-4

## Abstract

The global prevalence of osteoporosis is rising, particularly among the elderly and post-menopausal population. Although natural flavonoids can inhibit osteoclast overactivation, their low abundance and extraction challenges limit clinical translation. In this study, we synthesized a flavonoid derivative, SZQ-4, and evaluated its therapeutic potential for post-menopausal osteoporosis (PMO). Using an RANKL-induced osteoclastogenesis model in vitro, we demonstrated through TRAP staining, RT-qPCR, and bone resorption assays that SZQ-4 significantly suppresses osteoclast formation and activity. Mechanistically, RNA-seq, Western blot, siRNA knockdown, and plasmid-based overexpression experiments revealed that SZQ-4 reduces RANKL-induced reactive oxygen species (ROS) production, regulates *SIRT3* expression, and improves mitochondrial function, thereby attenuating osteoclast differentiation. In an ovariectomy-induced bone loss mouse model, SZQ-4 treatment markedly alleviated femoral bone loss, decreased osteoclast numbers, and lowered ROS levels in the bone marrow microenvironment. Collectively, our findings indicate that SZQ-4 inhibits osteoclast-driven bone resorption by modulating the ROS-*SIRT3*–mitochondrial function axis, highlighting its potential as a candidate for preventing pathological bone loss.

## 1. Introduction

Osteoporosis, a prevalent metabolic skeletal disorder characterized by compromised bone microarchitecture and reduced bone mass, results in increased bone fragility and susceptibility to fractures, particularly at weight-bearing sites such as the hip and vertebrae [1]. This condition represents a significant public health burden, contributing substantially to disability, mortality, and healthcare expenditures, especially among elderly and postmenopausal populations [2,3]. In China alone, projections indicate that osteoporosis-related fractures will reach 5.99 million annual cases with associated costs soaring to $25.43 billion by 2050 [4], underscoring the urgent need for effective preventive strategies. The pathophysiology of osteoporosis fundamentally disrupts the physiological balance of bone remodeling, a tightly regulated process that maintains skeletal integrity through the coordinated activities of bone-forming osteoblasts and bone-resorbing osteoclasts [5,6]. Excessive osteoclastic bone resorption unopposed by adequate osteoblastic bone formation constitutes the primary mechanism underlying progressive bone loss and the development of osteoporosis [7,8].

Osteoclasts, multinucleated giant cells originating from hematopoietic monocyte/macrophage precursors [9], undergo a complex differentiation process initiated by macrophage colony-stimulating factor (M-CSF) and receptor activator of nuclear factor kappa-B ligand (RANKL) signaling [10]. This differentiation requires substantial bioenergetic support [11], with mitochondria serving as crucial organelles that generate adenosine triphosphate (ATP) through oxidative phosphorylation while simultaneously regulating reactive oxygen species (ROS) production, metabolic intermediate synthesis, and cellular stress responses [12]. During RANKL-induced osteoclastogenesis, mitochondrial biogenesis is significantly enhanced to meet the increased energy requirements [13]. Notably, mitochondrial complex I deficiency redirects cellular metabolism from fatty acid oxidation toward glycolysis, thereby suppressing osteoclast differentiation and bone-resorptive capacity [14]. This positions mitochondrial function as a promising therapeutic target for the intervention of osteoporosis.

The sirtuin family, particularly the mitochondrial-localized *SIRT3*, has emerged as a critical regulator of cellular metabolism and mitochondrial homeostasis [15,16]. *SIRT3*, an NAD+-dependent deacetylase, maintains mitochondrial integrity through regulation of metabolic pathways, gene expression, and genomic stability [17,18]. Its involvement in age-related pathologies, including neurodegenerative disorders, cardiovascular diseases, and skeletal conditions [19,20], highlights its broad physiological significance. In bone metabolism, *SIRT3* modulates multiple processes, including mesenchymal stem cell aging, osteoblast differentiation, osteoclastogenesis, and maintenance of bone mineral density [21]. Experimental evidence demonstrates that *SIRT3* deficiency exacerbates osteoclast formation and accelerates bone loss in murine models [22], primarily by compromising the regulation of mitochondrial superoxide dismutase (SOD2). Crucially, SIRT3-mediated deacetylation of lysine 68 in SOD2 represents a key mechanism for restraining osteoclast differentiation [22], establishing *SIRT3* as a pivotal regulator of osteoclast mitochondrial function and a compelling therapeutic target for osteoporosis [23].

Current pharmacological management of osteoporosis remains suboptimal. While established therapies, including estrogen, calcitonin, bisphosphonates, and denosumab, demonstrate efficacy, their utility is constrained by significant adverse effects and potential toxicities [24,25]. Although estrogen replacement therapy and calcitonin can be used to prevent osteoporosis, long-term use of either may increase the risk of cancer [26,27]. Although calcium and vitamin D supplementation support bone health, their standalone efficacy in managing osteoporosis is limited [28]. These limitations necessitate the development of novel therapeutic agents with improved safety profiles.

Flavonoids, a class of bioactive polyphenolic compounds ubiquitous in plants and foods, are promising candidates due to their potent antioxidant properties and anti-resorptive effects [29,30,31]. Multiple flavonoid derivatives demonstrate the capacity to directly or indirectly suppress osteoclast differentiation and prevent bone loss [32]. However, the clinical translation of their natural extracts faces practical challenges, including complex extraction processes, limited oral bioavailability, and difficulties with large-scale, sustainable production, which have constrained their further development [33].

Advances in synthetic chemistry have enabled the development of flavonoid analogs with enhanced pharmaceutical properties. The fundamental flavonoid structure comprises 15 carbon atoms arranged as C6-C3-C6, forming two aromatic rings connected by a three-carbon bridge that typically cyclizes into a heterocyclic pyran ring [34,35]. Parallel developments in natural product chemistry have identified maleimide-containing compounds (general structure: -CO-N(R)-CO-) from fungal and marine sources as biologically active scaffolds [36]. Particularly, maleimide-fused carbazoles demonstrate remarkable osteogenic potential at submicromolar concentrations, potentially through glycogen synthase kinase inhibition and activation of the β-catenin pathway [37].

Building upon these structural insights, we previously developed chrome-maleimide heterocomplexes via Heck oxidation, culminating in SZQ-4—a synthetic flavonoid derivative exhibiting superior solubility, absorption kinetics, and chemical stability compared to natural analogs [38]. This compound was synthesized through ruthenium(II)-catalyzed direct coupling of maleimide and chromium at the C5 position. Given the established anti-osteoclastogenic properties of flavonoids, we therefore hypothesize that SZQ-4, a novel flavonoid–maleimide hybrid, attenuates osteoclast-driven bone loss by regulating *SIRT3* expression, thereby improving mitochondrial function and suppressing osteoclast differentiation in vitro and in vivo. This study aims to test this hypothesis using both cellular and ovariectomy-induced osteoporosis models, providing proof-of-concept for *SIRT3*-targeted pharmacological intervention in osteoporosis. Furthermore, we elucidate the molecular mechanisms underlying SZQ-4 action, with a particular focus on *SIRT3*-mediated regulation of mitochondrial function in osteoclasts.

## 2. Materials and Methods

### 2.1. Materials

The primary antibodies and reagents utilized in this study are detailed in Supplementary Table S1. SZQ-4 was synthesized by our research group, and its purity was confirmed to be greater than 95% by high-performance liquid chromatography (HPLC). The synthesis was catalyzed by [Ru(p-cymene)Cl_2_]_2_ (5 mol%) in the presence of AgNTf_2_ (20 mol%) and AgOAc (3 equiv.) at 120 °C. The reaction between chromones and maleimides was carried out in 1,2-dichloroethane (DCE, 2 mL), yielding the desired products in excellent yields. SZQ-4 was dissolved in DMSO to prepare a 10 mM stock solution.

### 2.2. Osteoclast Differentiation

Bone marrow-derived macrophages (BMDMs) were prepared from the femur and tibia bone marrow of 8-week-old male C57BL/6J mice provided by the Wenzhou Medical University Animal Center. C57BL/6 mice were given free access to standard chow and water at 20–24 °C. These BMDMs were then cultured in α-MEM with 10% FBS, penicillin, streptomycin, and M-CSF (50 ng/mL) (complete α-MEM). After a 3-day incubation, the attached cells were used for experiments. The RAW264.7 cell line was purchased from ATCC and maintained in DMEM supplemented with 10% FBS at 37 °C under 5% CO_2_. For subculture, cells were passaged by gentle pipetting when they reached approximately 80–90% confluence: the old medium was discarded, fresh complete medium was added, and cells were detached by gentle pipetting to form a single-cell suspension for subsequent seeding. For osteoclast differentiation assays, both BMDMs and RAW264.7 cells were placed into 96-well plates overnight at a density of 1 × 10^4^ cells/well. The following day, BMDMs were stimulated with M-CSF (50 ng/mL) and RANKL (30 ng/mL), while RAW264.7 cells were stimulated with RANKL (50 ng/mL) alone. Cells were treated in the presence or absence of increasing concentrations of SZQ-4 (0.25, 0.5, 1, 2, 5, and 10 μM). The medium was changed every 2 days until osteoclasts formed. Cells were then fixed with 4% paraformaldehyde, and tartrate-resistant acid phosphatase (TRAP) activity was detected using a leukocyte acid phosphatase kit. Osteoclasts were defined as cells with more than three nuclei.

### 2.3. Cytotoxicity Assay

BMDMs were seeded in 96-well plates at a density of 1 × 10^4^ cells/well. After incubation for 24 h, cells were treated with different concentrations of SZQ-4 for 48 h. At the end of the experiment, MTT solution and medium were mixed in a 1:4 ratio in the well plate and incubated for 4 h. Optical density was measured at 490 nm using a microplate reader (BMG LABTECH, Ortenberg, Germany).

### 2.4. Real-Time (RT)-PCR Analysis

BMDMs were inoculated into 6-well plates at a density of 3.5 × 10^5^ cells/well. The cells were grouped and treated as described above. Total RNA was extracted using TRIzol (Invitrogen, Waltham, MA, USA): cells were lysed in 1 mL TRIzol, mixed with 200 μL chloroform, centrifuged at 12,000× *g* for 15 min at 4 °C; the aqueous phase was precipitated with isopropanol, washed with 75% ethanol, and dissolved in DEPC-treated water. RNA concentration and purity were assessed with a NanoDrop 2000 (Thermo Fisher Scientific, Waltham, MA, USA); samples with A260/A280 ratios of 1.9–2.1 and A260/A230 > 2.0 were used. For reverse transcription, 1 μg RNA was reverse-transcribed using HiScript III RT SuperMix (+gDNA wiper) (Vazyme, Nanjing, China) with gDNA elimination (42 °C, 2 min), reverse transcription (50 °C, 15 min), and inactivation (85 °C, 5 s). No-RT controls were included. cDNA was stored at −20 °C. qPCR was performed in triplicate in 20 μL reactions containing ChamQ Universal SYBR qPCR Master Mix (Vazyme, China), 0.4 μM primers, and 1:5 diluted cDNA on a StepOne™ System (Applied Biosystems, Waltham, MA, USA) with cycling: 95 °C for 30 s, then 40 cycles of 95 °C for 10 s and 60 °C for 30 s, followed by melt curve analysis. Primer sequences (Table S2) were exon-spanning and validated for efficiencies of 90–110%. No-template controls were included. Relative expression was calculated using the 2^−ΔΔCt^ method with β-actin as reference; its stability was confirmed.

### 2.5. F-Actin Ring Formation Assay

BMDMs were seeded in 24-well plates at a density of 5 × 10^4^ cells/well. Cells were grouped and treated as described above. Mature osteoclasts were fixed with 4% formaldehyde solution, permeabilized with 0.5% Triton-X100 solution, and incubated with TRITC marker ghost peptide and DAPI in the dark. The morphology of the F-actin ring was observed using a multifunctional inverted fluorescence microscope (Axio Observer A1, Zeiss, Jena, Germany). The coverage of multinucleated osteoclasts was determined using Image-Pro Plus software (V 6.0).

### 2.6. Mitochondria Functional and Morphological Imaging Assays

BMDMs were seeded at a density of 1 × 10^4^ cells/well in 96-well plates. Cells were grouped and treated as described above for osteoclast differentiation. mtROS production was assessed using the mitochondria-targeted probe MitoSOX. Images were captured using an inverted fluorescence microscope. Excitation and emission wavelengths were 357/410 nm for MitoSOX, 548/574 nm for TMRM, and 491/513 nm for MitoGreen. TMRM was used at a working concentration of 100 nM, and cells were incubated with the dye for 10 min at 37 °C. After incubation, cells were washed three times with pre-warmed PBS (37 °C) to remove excess dye. The average fluorescence intensity was analyzed using ImageJ software (version 1.54).

### 2.7. Adenosine Triphosphate (ATP) Detection Assay

BMDMs were seeded in 96-well plates at a density of 1 × 10^4^ cells/well. Cells were grouped and treated as described above for osteoclast differentiation. ATP production was detected using a CellTiter-Glo Assay. Approximately 100 µL of reagent was added to each well, and the contents were mixed. The plate was then incubated in the dark on a horizontal shaker for 5 min, followed by a 10 min standing period at 25 °C. The chemiluminescent signal (RLU) was subsequently recorded using a microplate reader. To normalize ATP levels, total protein concentration was measured using a bicinchoninic acid (BCA) protein assay kit (Thermo Fisher Scientific, USA) according to the manufacturer’s instructions. Briefly, cell lysates were mixed with the BCA working reagent and incubated at 37 °C for 30 min. Absorbance was measured at 562 nm using a microplate reader. Protein concentrations were calculated from a standard curve generated with bovine serum albumin (BSA).

### 2.8. Bone Resorption Assays

BMDMs were inoculated into 48-well bone resorption assay plates coated with artificial bone slices at a density of 3 × 10^4^ cells/well. The bone resorption assay was performed using the Bone Resorption Assay Kit 48 (Cat. No. BRA-48KIT; Cosmo Bio Co., Ltd., Tokyo, Japan). Cells were grouped and induced for osteoclast differentiation as described above, and maintained in the respective culture medium for 6 days. Mature osteoclasts were then lysed with PBS containing 0.1% Triton X-100 on ice for 10 min to disrupt the cell membrane. The bone slices were subsequently rinsed with 0.5% sodium hypochlorite, washed thoroughly with distilled water, and air-dried. Resorption pit formation on the bone slices was observed and imaged using an SMZ800N stereomicroscope (Nikon, Tokyo, Japan). For each group, at least five wells were analyzed, with three randomly selected non-overlapping fields per well. Images were quantified using ImageJ software, and analyses were performed in a blinded manner.

### 2.9. Total Antioxidant Capacity Assay

BMDMs were seeded in 96-well plates at a density of 1 × 10^4^ cells/well. Cells were grouped and treated as described above. Approximately 200 μL of ABTS working solution was added to each well of a 96-well plate. Then, 10 μL of various samples were added to the wells and gently mixed. After incubating at room temperature for 2–6 min, A734 was measured, and the total antioxidant capacity of the samples was calculated from the standard curve.

### 2.10. ROS Assay

BMDMs were seeded in 96-well plates at a density of 1 × 10^4^ cells/well. Cells were grouped and treated as described above. Intracellular ROS levels were also measured using the 2′,7′-dichlorodihydrofluorescein diacetate (H2DCFDA, Thermo Fisher Scientific, Waltham, MA, USA). Following treatment, cells were incubated with DCFH-DA for 20 min at 37 °C, observed using an inverted fluorescence microscope, and measured at 488 nm excitation and 525 nm emission using a fluorescence spectrophotometer.

### 2.11. DPPH-Radical Scavenging Assay

BMDMs were seeded in 96-well plates at a density of 1 × 10^4^ cells/well. Cells were grouped and treated as described above. According to the manufacturer’s instructions, the scavenging activity of the 2,2-diphenyl-1-picrylhydrazyl (DPPH) radical (Solarbio, Beijing, China) was used to determine antioxidant properties. Antioxidant activity was calculated and expressed as a percentage.

### 2.12. Mitochondrial Oxygen Respiration Measurement

RAW264.7 cells were seeded in 6-well plates at a density of 3.5 × 10^5^ cells/well. Cells were grouped and treated as described above. On the fourth day of culture, cells were digested and resuspended for mitochondrial function analysis. The mitochondrial oxygen consumption rate (OCR) was measured using an Oxygraph-2K (O2K) instrument (Oroboros Instruments GmbH, Innsbruck, Austria), with sequential injection of oligomycin and carbonyl cyanide-4-(trifluoromethoxy) phenylhydrazone (FCCP) at final working concentrations of 1 μM and 2 μM, respectively.

### 2.13. RNA-Seq

Total RNA was extracted from BMDMs treated with RANKL alone or in combination with SZQ-4 (*n* = 3 per group) using TRIzol reagent. RNA integrity was assessed using the RNA Nano 6000 Assay Kit on a Bioanalyzer 2100 system (Agilent Technologies, Santa Clara, CA, USA). Sequencing libraries were prepared following the standard protocol for the NEBNext Ultra RNA Library Prep Kit for Illumina (Ipswich, MA, USA): mRNA was purified, fragmented, and reverse-transcribed into cDNA. After end repair, adenylation, and adapter ligation, cDNA fragments of approximately 370–420 bp were selected using the AMPure XP system (Beckman Coulter, Brea, CA, USA) and enriched by PCR. Library quality was verified on the Agilent Bioanalyzer 2100 system. Indexed libraries were clustered on a cBot Cluster Generation System and sequenced on an Illumina NovaSeq 6000 platform to generate 150 bp paired-end reads. Raw reads were processed with fastp to obtain clean reads by removing adapter sequences and low-quality bases. Quality metrics, including Q20, Q30, and GC content, were calculated. Clean reads were then aligned to the mouse reference genome (GRCm39) using HISAT2 (v2.0.5). Gene expression levels were quantified as FPKM using featureCounts (v1.5.0-p3). Differential expression analysis between the two groups was performed with DESeq2 (v1.20.0) in R, applying an adjusted *p*-value < 0.05 and |log_2_FoldChange| > 1 as significance thresholds. Gene Ontology (GO) and Kyoto Encyclopedia of Genes and Genomes (KEGG) pathway enrichment analyses of differentially expressed genes were conducted using the clusterProfiler R package (version 4.10.0). To specifically examine mitochondrial function, enrichment analysis was also performed using the MitoCarta database as a reference gene set. The raw RNA sequencing data have been deposited in the NCBI Sequence Read Archive (SRA) under BioProject accession number PRJNA1428510.

### 2.14. Plasmid Interference and Cell Transfection

RAW264.7 cells were seeded in 24-well plates at a density of 5 × 10^4^ cells/well. Transfection was performed after the cells had proliferated to 50% confluence. For *SIRT3* knockdown experiments, siRNA targeting *SIRT3* was used, with a non-specific siRNA (si-control) serving as the negative control. For *SIRT3* overexpression, the pcDNA3.1-m*SIRT3* plasmid was employed, with an empty vector (vector-EGFP) used as the corresponding control. For transfection, Lipofectamine 3000 and Opti-MEM were added to tube A at a dilution of 1:200 and mixed thoroughly. In tube B, the siRNA or plasmid was diluted with Opti-MEM to prepare a premix; P3000 was then added to the plasmid mixture at a 1:1 ratio and mixed completely. Tubes A and B were incubated separately for 5 min. The two solutions were combined, mixed gently, and allowed to stand at room temperature for 15 min. The resulting plasmid– or siRNA–liposome complex was added to the cells, which were incubated for 48 h at 37 °C and subsequently treated according to their experimental groups. Detailed information regarding the *Sirt3* plasmid construct is provided in Table S3.

### 2.15. Western Blot (WB) Analysis

Each sample group was separated by sodium dodecyl sulfate–polyacrylamide gel electrophoresis (SDS–PAGE) and subsequently transferred onto a polyvinylidene fluoride (PVDF) membrane (Millipore, USA). Prior to transfer, PVDF membranes were activated with methanol. Protein transfer was performed using a wet transfer system. After transfer, the membranes were blocked with 5% skim milk dissolved in Tris-buffered saline containing 0.1% Tween-20 (TBST) for 1 h at room temperature to prevent nonspecific binding. The membranes were then incubated overnight at 4 °C with the following primary antibodies: anti-SIRT3 (1:1000, Cell Signaling Technology, Danvers, MA, USA), anti-NFATc1 (1:1000, Cell Signaling Technology), and β-actin (1:8000, Proteintech, Rosemont, IL, USA). After primary antibody incubation, the membranes were washed three times with TBST (5–10 min each) and then incubated with appropriate horseradish peroxidase (HRP)-conjugated secondary antibodies for 1 h at room temperature. Following additional washes with TBST, protein bands were visualized using an enhanced chemiluminescence (ECL) detection system (Bio-Rad, Hercules, CA, USA). The band intensities were quantified using ImageJ software (National Institutes of Health, Bethesda, MD, USA), and the expression levels of target proteins were normalized to β-actin.

### 2.16. Animal Study Design

Eight-week-old female C57BL/6 mice were maintained in an independent ven-tila-tion system under a 12 h light/12 h dark cycle. All animal experimental protocols were approved by the Animal Ethics Committee of Wenzhou Medical University (xmsq2025-0418). All mice were divided into three groups, with five ani-mals in each group: a sham operation group (Sham + Vehicle), a solvent group (OVX + Vehicle), and an SZQ-4 group (OVX + SZQ-4). Mice in the OVX groups under-went surgery via a lateral retroperitoneal approach with ketamine/xylazine anesthesia, while the sham group underwent a mock operation. SZQ-4 was first dissolved in DMSO and then diluted with saline to 0.4 mg/mL following a 1000-fold dilution. The concentration of DMSO was 0.1%, a safe concentration. The sham operation and sol-vent groups were administered 0.5 mL of solvent via gavage, while the SZQ-4 group received 10 mg/kg of SZQ-4 suspen-sion via gavage once every 2 days for 8 weeks. The detailed process of the ovarian re-section experiment is as follows: female mice (C57BL/6J, 8 weeks old) were anesthetized. Bilateral dorsolateral incisions expose the abdominal cavity. Ovaries are exteriorized, and the surrounding vasculature ligated. Sham controls undergo an identical lapa-rotomy without ovary removal. Inci-sions are sutured, and postoperative penicillin is administered for 72 h. The effect of bone loss in the animal model can be obtained by comparing sham + vehicle and OVX + vehicle. During the feeding process, the weight of each mouse was measured weekly, and the dosage was adjusted. After feeding for 8 weeks, all mice were eu-thanized via cervical dislocation under anesthesia. Each mouse’s femur and major or-gans were collected, placed in embedding boxes, marked, and fixed in a 4% paraform-aldehyde solution.

### 2.17. Micro-Computed Tomography (Micro-CT) Detection

The bone tissue and right femur were scanned using a Skyscan 1176 micro-CT instrument (Bruker Micro-CT, Kontich, Belgium). Scanning was performed with a voxel size of 9 μm at an energy of 55 kVp and a current of 145 μA, and a 0.5 mm aluminum filter was applied in accordance with the manufacturer’s protocol to reduce beam-hardening artifacts. Images were subsequently reconstructed using Nrecon software (version 2.0). The trabecular bone region of interest was manually defined, and bone parameters were determined by binarizing the trabecular bone with a constant threshold. Using the CTAn program (version 2.0), bone volume (BV/TV), trabecular number (Tb.N), connective density (Conn.Dn), and trabecular thickness (Tb.Th) were analyzed.

### 2.18. Histomorphometric Analysis

After fixation, the right femur was decalcified in 10% EDTA (pH 7.4) at 4 °C for 4 weeks, with the decalcification solution replaced weekly. Completion of decalcification was confirmed using the needle penetration method prior to further tissue processing, dehydrated through a graded ethanol series, cleared in xylene, and embedded in paraffin. Sections (4 μm) were stained with hematoxylin and eosin (H&E), and TRAP activity was assessed using a TRAP staining kit (Sigma-Aldrich, St. Louis, MO, USA, Catalog No. 387A) to observe the OCs in each visual field. In the proximal tibia at ×200 magnification, the trabecular structure (trabecular volume, bone surface, and quantity) in H&E-stained sections was measured using morphometry. Fifteen to twenty visual fields were counted, and the area was measured with ImageJ software. The major organ sections of the heart, liver, spleen, lung, and kidney was stained with H&E.

### 2.19. Femoral Immuno-Fluorescence Staining

Tissue antigen sections were subjected to antigen retrieval using 0.25% trypsin-EDTA for 20 min, followed by blocking with 5% goat serum for 30 min. The primary antibodies, rabbit anti-SIRT3 (1:300) and anti-CD68 (1:200, Immunoway, San Jose, CA, USA), were then applied and incubated at 4 °C overnight. After rewarming to 25 °C, the species-matched Alexa Fluor 488– or Alexa Fluor 594–conjugated goat anti-rabbit or anti-mouse IgG secondary antibodies (Beyotime Biotechnology, Shanghai, China) were applied and incubated for 1 h in the dark. Finally, the sections were mounted with an anti-fade mounting medium containing DAPI. Images were acquired using a fluorescence microscope and analyzed statistically.

### 2.20. ROS Measurements In Vivo

Mice received an intravenous injection of DHE (10 mg/kg) 24 h before euthanasia. The DHE solution was prepared by dissolving 25 mg of DHE powder in 250 μL of DMSO, followed by sonication to ensure complete dissolution. This stock solution was then diluted 100-fold with saline immediately prior to use. Left femurs were decalcified, embedded, and sectioned into 10 μm frozen sections after fixation. Following hydration with PBS for 5 min and permeabilization with 0.3% Triton X-100 in the dark for 10 min, the samples were mounted with an anti-fade mounting medium containing DAPI and imaged using a fluorescence microscope for subsequent statistical analysis. The region of interest (ROI) was defined as the area adjacent to the growth plate in the distal femur, manually outlined using ImageJ software. Mean fluorescence intensity was measured after background subtraction, which was performed by subtracting the mean intensity of a cell-free area from each ROI. To account for variations in tissue area or cell density, fluorescence values were normalized to the corresponding DAPI-positive nuclear area within the same ROI. All quantifications were performed blindly with respect to experimental groups to avoid observer bias.

### 2.21. Statistical Analysis

All data are presented as the mean ± standard deviation (SD). Differences among multiple groups were analyzed by one-way analysis of variance (ANOVA) followed by Tukey’s honestly significant difference (HSD) post hoc test for pairwise multiple comparisons. Statistical analyses were performed using GraphPad Prism 8.0. Significance levels are denoted as * *p* < 0.05, ** *p* < 0.01, *** *p* < 0.001, and NS (not significant).

## 3. Results

### 3.1. SZQ-4 Suppresses RANKL-Induced Osteoclastogenesis In Vitro

**Figure 1:**
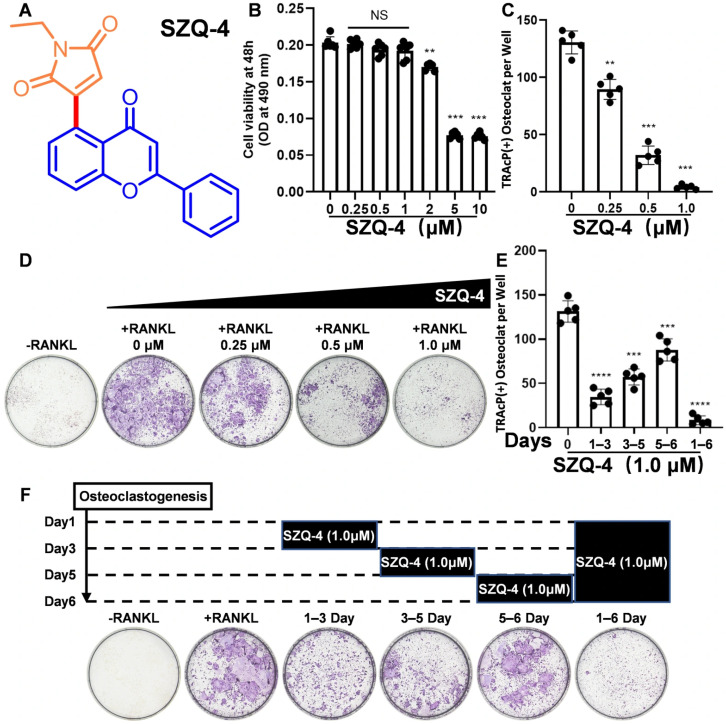
SZQ-4 suppressed RANKL-induced osteoclastogenesis in vitro. (**A**) Chemical structure and formula of SZQ-4. (**B**) Cell viability of BMDMs after co-culture with various SZQ-4 concentrations (*n* = 5 independent samples). (**C**) Quantification of TRAP-positive multinucleated cells after co-culture with various SZQ-4 concentrations (*n* = 5 independent samples). (**D**) Representative TRAP staining images of BMDMs show that SZQ-4 inhibited osteoclastogenesis in a dose-dependent manner. (**E**) Quantification of TRAP-positive multinucleated cells treated with SZQ-4 during different periods (*n* = 5 independent samples). (**F**) Representative TRAP staining images of BMDMs after treatment with SZQ-4 for the indicated days during osteoclastogenesis. NS adjusted *p* > 0.05; ** adjusted *p* < 0.01; *** adjusted *p* < 0.001; **** adjusted *p* < 0.0001; error bars = SD; data are presented as mean values ± SD.

Figure 1A shows the chemical structure of SZQ-4. Based on the Heck-type oxidative pathway, chromones and maleimides were dissolved in Ru to obtain SZQ-4. Notably, SZQ-4 did not inhibit BMDMs’ proliferation across a concentration range of 0–1.0 μM (Figure 1B). To elucidate the effect of SZQ-4 on osteoclast formation, BMDMs were cultured in 96-well plates and treated with M-CSF and RANKL in the presence or absence of various concentrations of SZQ-4. Numerous TRAP-positive multinucleated osteoclasts were formed in the RANKL-induced control group (without SZQ-4), whereas increasing concentrations of SZQ-4 inhibited osteoclastogenesis in a dose-dependent manner (Figure 1C,D). Although SZQ-4 treatment at various stages of osteoclast differentiation consistently inhibited osteoclast differentiation, the effects were most pronounced during the early phase. None of these short-term treatments was as effective as prolonged administration (Figure 1E,F).

### 3.2. SZQ-4 Inhibits Osteoclast-Specific Gene Expression, Podosome Belt Formation, and Inhibits Osteoclast Resorptive Function

**Figure 2:**
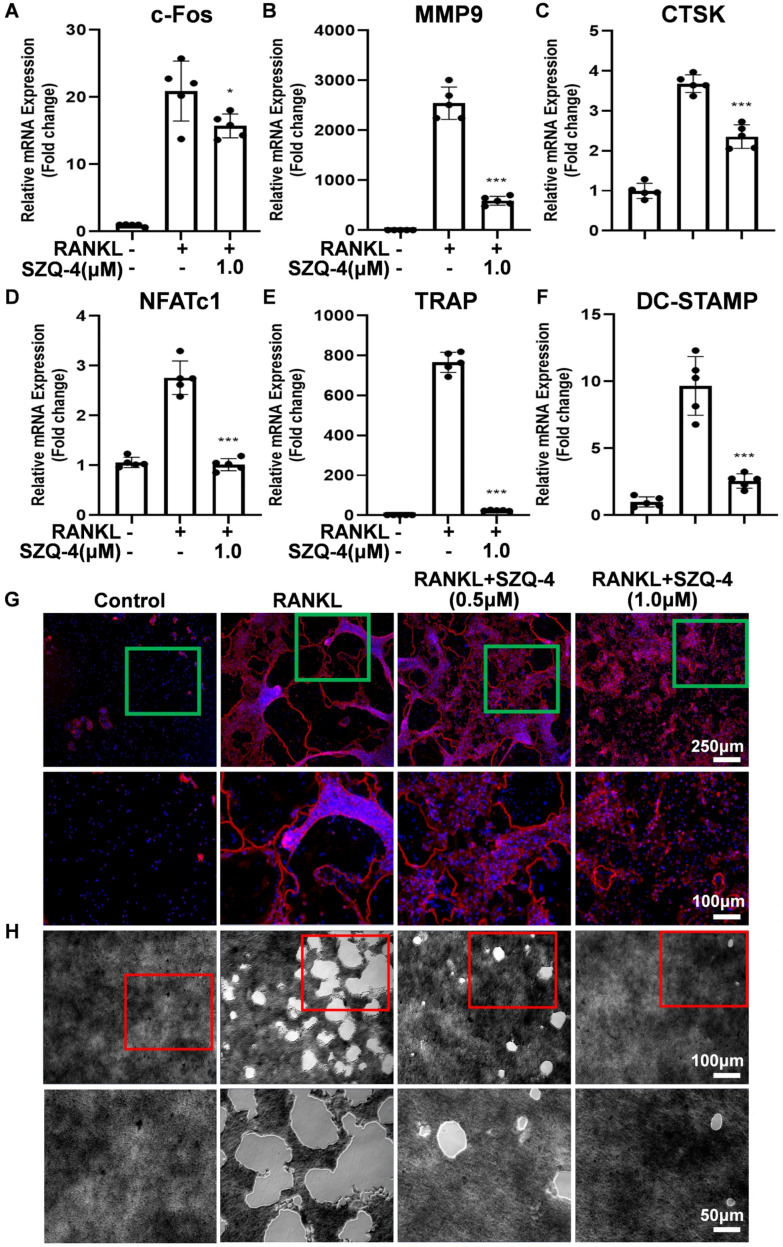
SZQ-4 inhibited osteoclast-specific gene expression, podosome belt formation, and osteoclast resorptive function. (**A**–**F**) qPCR analysis of osteoclast-specific gene expression of *c*-*Fos*, *MMP9*, *CTSK*, *NFATc1*, *TRAP*, and *DC*-*STAMP* in BMDMs stimulated with RANKL for 3 d in the presence of SZQ-4 (*n* = 5 independent samples). (**G**) Representative fluorescence images of podosome belt formation. (**H**) Representative images of hydroxyapatite resorption (*n* = 5 independent samples). * adjusted *p* < 0.05, *** adjusted *p* < 0.001; error bars = SD; data are presented as mean values ± SD.

When osteoclast differentiation is induced, several osteoclast-specific genes, including *c*-*Fos, MMP9*, *CTSK*, *NFATc1*, *TRAP*, and *DC*-*STAMP*, are upregulated in BMDMs. Quantitative PCR was used to monitor the expression of these genes and assess the effect of SZQ-4 on osteoclast-specific gene expression. The corresponding gene expression was inhibited during RANKL-induced osteoclastogenesis following the addition of SZQ-4 (Figure 2A–F). These results confirmed that SZQ-4 suppressed the expression of osteoclast-specific genes, thereby inhibiting osteoclastogenesis in vitro. Mature osteoclast cells were co-stained with rhodamine-phalloidin to observe podosome band formation and morphological changes in cells treated with or without SZQ-4. Figure 2G shows podocorpuscle bands with intact nuclei and well-defined boundaries formed in mature osteoclasts after RANKL stimulation. The number of osteoclasts on the pore plates, with F-actin (red) and nuclei (blue), decreased progressively with SZQ-4 treatment. Figure 2H presents representative images of hydroxyapatite resorption, showing a reduction in resorption area following SZQ-4 treatment (Figure S1).

### 3.3. SZQ-4 Exhibits Concentration-Dependent Antioxidant Activities and Regulates Mitochondrial Function in Osteoclasts

**Figure 3:**
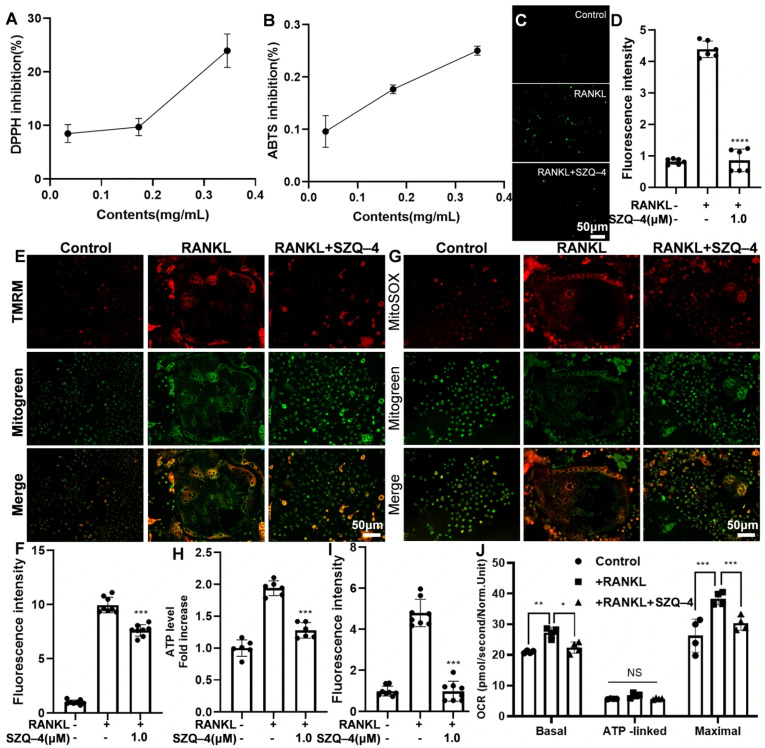
SZQ-4 exhibits concentration-dependent antioxidant activities and regulates mitochondrial function in osteoclasts. (**A**) DPPH-radical scavenging activity (*n* = 3 independent samples). (**B**) ABTS radical-scavenging activity (*n* = 3 independent samples). (**C**) Representative DCF staining images show that SZQ-4 inhibited intracellular ROS. (**D**) Quantification of fluorescence intensity for DCF staining images (*n* = 5 independent images). (**E**) Representative TMRM fluorescence staining images of mitochondrial membrane potential. (**F**) Quantification of MMP fluorescence intensity (*n* = 5 independent images). (**G**) Representative MitoSOX staining images of mtROS. (**H**) ATP level in RANKL-induced osteoclastogenesis. (**I**) Quantification of mtROS fluorescence intensity (*n* = 5 independent samples). (**J**) OCR level of osteoclasts induced by RANKL (*n* = 4 independent samples). * adjusted *p* < 0.05, ** adjusted *p* < 0.01, *** adjusted *p* < 0.001, **** adjusted *p* < 0.0001; error bars = SD; data are presented as mean values ± SD.

To investigate whether SZQ-4 reduces ROS production during osteoclast differentiation, we first assessed its antioxidant capacity using DPPH and ABTS radical-scavenging assays. As shown in Figure 3A,B, the radical-scavenging activity of SZQ-4 increased in a concentration-dependent manner. Intracellular ROS levels were visualized using the cell-permeable oxidation-sensitive dye H2DCFDA. Figure 3C,D demonstrate that after treatment with SZQ-4 at a concentration of 1.0 µM, the fluorescence intensity of DCF decreased in a dose-dependent manner when M-CSF and RANKL stimulated BMDMs. Simultaneous staining with MitoSOX and MitoGreen can target mitochondria and reflect mitochondrial ROS intensity. Interestingly, MitoSOX staining showed that SZQ-4 treatment reduced mtROS levels during osteoclast differentiation (Figure 3G,I).

Mitochondrial membrane potential was detected via TMRM fluorescence staining. After SZQ-4 treatment, TMRM fluorescence intensity decreased significantly. These results indicate that SZQ-4 reduced the high mitochondrial membrane potential induced by RANKL (Figure 3E,F). Simultaneously, SZQ-4 reduced ATP levels during RANKL-induced osteoclastogenesis (Figure 3H). Cellular OCR measurements are key indicators of cellular metabolic function. Figure 3J shows that SZQ-4 inhibited the RANKL-induced increase in OCR levels in osteoclasts. These results indicate that SZQ-4 inhibits osteoclast formation and activity by regulating mitochondrial function.

### 3.4. SIRT3 Plays a Significant Role in the Inhibition of Osteoclast Differentiation by SZQ-4

**Figure 4:**
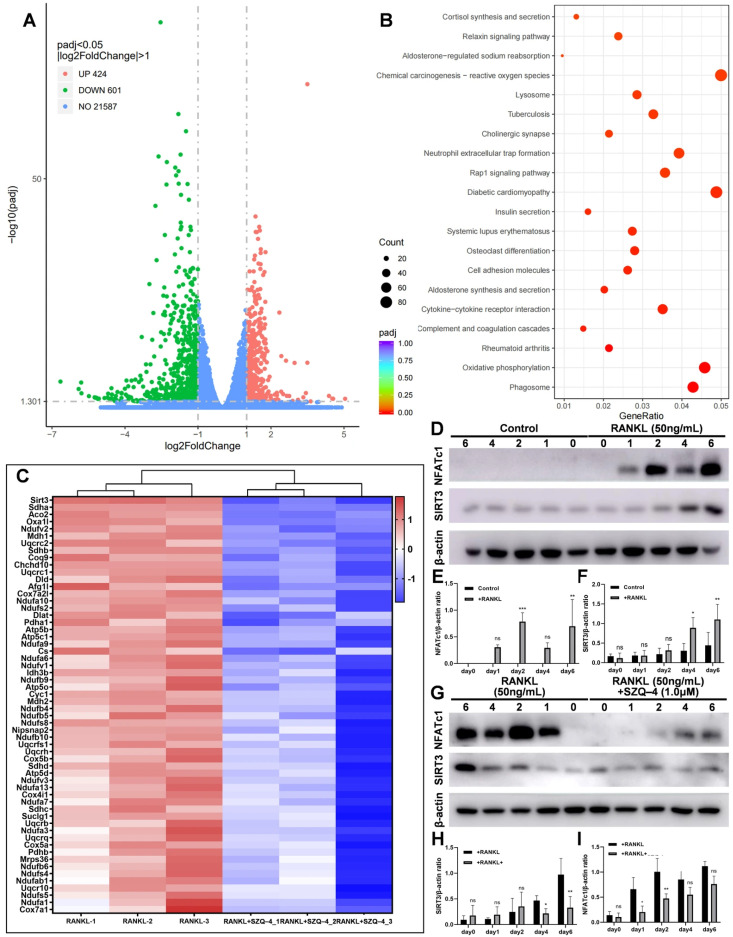
*SIRT3* plays a significant role in the inhibition of osteoclast differentiation by SZQ-4. (**A**) Volcano maps show the number of upregulated and downregulated genes. The dashed vertical lines indicate the thresholds of log2(Fold Change) = ±1, and the horizontal dashed line indicates the significance threshold (adjusted *p* = 0.05). (**B**) GO enrichment analysis revealed enriched signaling pathways. (**C**) Heatmaps showing differentially expressed genes. (**D**–**F**) WB images and the quantitative analysis of *SIRT3* expression levels in osteoclast differentiation with or without RANKL induction. (**G**–**I**) WB images and the quantitative analysis of *SIRT3* expression levels in osteoclast differentiation with or without SZQ-4 treatment. *n* = 3 independent samples, * adjusted *p* < 0.05, ** adjusted *p* < 0.01, *** adjusted *p* < 0.001, ns, not significant; error bars = SD; data are presented as mean values ± SD.

To determine the specific mechanism by which SZQ-4 inhibits osteoclast differentiation, RNA-seq was performed on RANKL-treated and SZQ-4 + RANKL-treated osteoclasts. The volcano map in Figure 4A shows 424 upregulated and 601 downregulated genes. GO enrichment analysis revealed strong enrichment of oxidative phosphorylation pathway genes after SZQ-4 treatment (Figure 4B). Since this study focused on the role of mitochondria in inhibiting SZQ-4 during osteoclast differentiation, genes associated with the enriched oxidative phosphorylation pathway were analyzed against the MitoCarta3.0 database. The heatmaps in Figure 4C show that *SIRT3* was differentially expressed. To validate the results obtained through RNA-seq, WB analysis was used to measure the expression levels of *SIRT3* and *NFATc1* at different time points (0, 1, 2, 4, and 6 d) of osteoclast differentiation after treatment with or without SZQ-4. *SIRT3* expression increased gradually with increasing treatment time during osteoclast differentiation (Figure 4D–I). Following SZQ-4 treatment, *SIRT3* expression was reduced compared to the untreated group. This might be the direct effect of the drug, or it might reflect the overall effect after osteoclast differentiation has been inhibited.

### 3.5. SZQ-4 Inhibits Osteoclast Differentiation Through SIRT3

**Figure 5:**
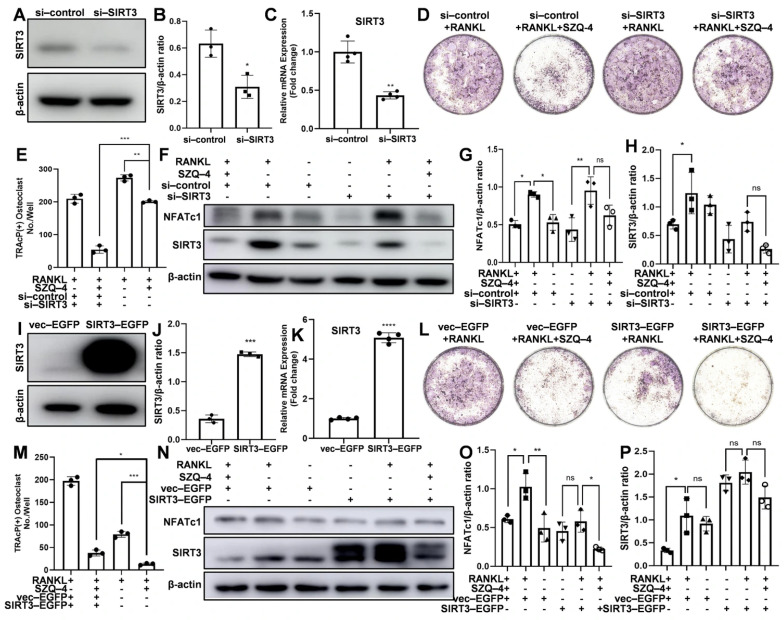
Knockdown or overexpression of *SIRT3* influenced osteoclast differentiation and affected the inhibitory effect of SZQ-4. (**A**,**B**) WB images and quantitative analysis of *SIRT3* protein expression levels (*n* = 3 independent samples). (**C**) *SIRT3* gene expression levels (*n* = 4 independent samples). (**D**,**E**) Representative images of TRAP staining and quantitative analysis of RAW264.7 cells treated with si-control or si-*SIRT3*, followed by osteoclast differentiation medium with or without SZQ-4 (*n* = 3 independent samples). (**F**–**H**) WB images and quantification of *SIRT3* and *NFATc1* protein levels in RAW264.7 cells treated with si-control or si-*SIRT3* and osteoclast induction medium with or without SZQ-4 (*n* = 3 independent samples). (**I**,**J**) WB images and quantification of *SIRT3* protein levels following treatment with vector-EGFP or *SIRT3*-EGFP (*n* = 3 independent samples)*.* (**K**) qRT-PCR analysis of *SIRT3* mRNA levels in cells with vector-EGFP or *SIRT3*-EGFP (*n* = 4 independent samples). (**L**,**M**) Representative TRAP staining images and quantitative analysis of RAW264.7 cells treated with vector-EGFP or *SIRT3*-EGFP, followed by osteoclast induction medium with or without SZQ-4 (*n* = 3 independent samples). (**N**–**P**) WB images and quantitative analysis of *SIRT3* and *NFATc1* protein expression levels after treatment with vector-EGFP or *SIRT3*-EGFP, and osteoclast induction medium with or without SZQ-4 (*n* = 3 independent samples). * adjusted *p* < 0.05, ** adjusted *p* < 0.01, *** adjusted *p* < 0.001, **** adjusted *p* < 0.0001, ns, not significant; error bars = SD; data are presented as mean values ± SD.

Figure 5A–C show that cells transfected with si-*SIRT3* showed lower *SIRT3* expression levels than those transfected with-their si-control counterparts. Figure 5D,E show that after the cells were transfected with si-control and treated with osteoclast induction medium containing SZQ-4, the number and volume of osteoclasts were significantly lower than those in the group treated with osteoclast induction medium alone. After the cells were transfected with si-*SIRT3* and treated with osteoclast induction medium, the number of differentiated osteoclasts was higher than that in cells transfected with si-control. Additionally, the inhibitory effect in cells transfected with SZQ-4 was less than that observed in cells transfected with si-control. Figure 5F–H show that the WB results corresponded to the results of TRAP staining. Compared to the si-control, the expression of *NFATc1* increased after si-*SIRT3* treatment; however, the inhibitory effect of SZQ-4 was weakened.

Figure 5I–K show that the mRNA and protein expression levels of *SIRT3* in cells transfected with *SIRT3*-EGFP were higher than those in cells transfected with vector-EGFP. After the cells were transfected with vector-EGFP and treated with osteoclast induction medium containing SZQ-4, the number and volume of osteoclasts were significantly smaller than those in the group treated with osteoclast induction medium alone (Figure 5L,M). After the cells were transfected with *SIRT3*-EGFP and treated with osteoclast induction medium, the number of differentiated osteoclasts was lower than that in cells transfected with vector-EGFP. Additionally, the inhibitory effect of the cells transfected with SZQ-4 was greater than that of those transfected with vector-EGFP. *NFATc1* expression decreased after *SIRT3*-EGFP treatment compared to vector-EGFP, with an enhanced inhibitory effect by SZQ-4 (Figure 5N–P).

### 3.6. SZQ-4 Regulates Mitochondrial Function Through SIRT3

**Figure 6:**
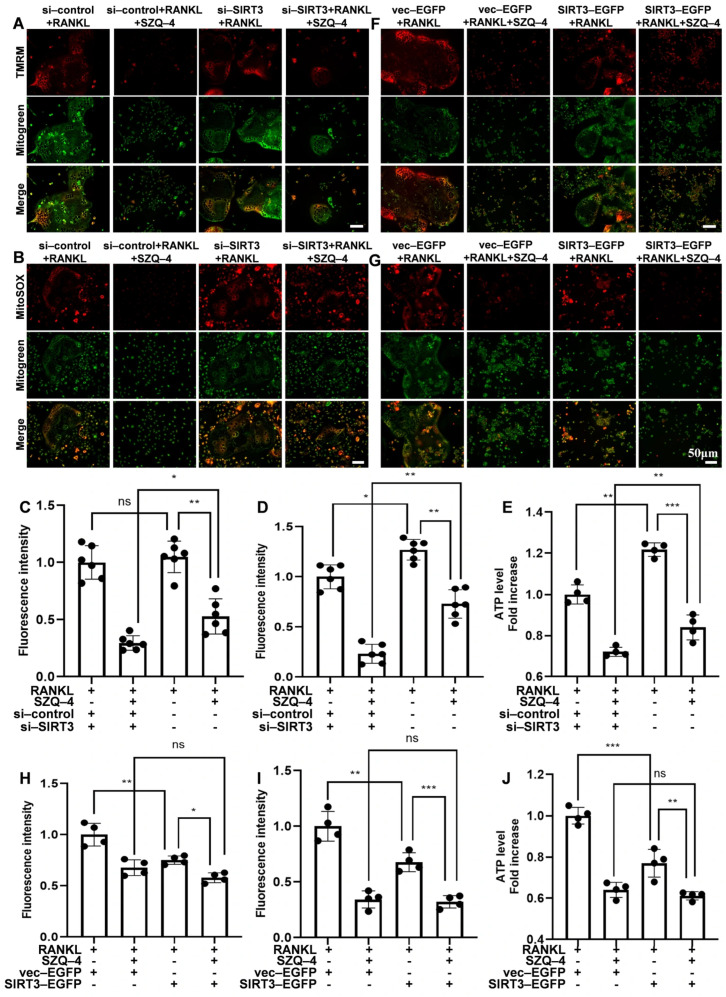
SZQ-4 regulates mitochondrial function through *SIRT3*. (**A**) Representative TMRM and (**B**) MitoSOX staining images of osteoclasts (*n* = 5 independent images). Quantitative analysis of (**C**) mitochondrial membrane potential, (**D**) ROS generation, and (**E**) ATP levels in osteoclasts after treatment with *s*i-control or si-*SIRT3*. (**F**) Representative TMRM staining and (**G**) MitoSOX staining images of osteoclasts (*n* = 5 independent images). Quantitative analysis of (**H**) mitochondrial membrane potential, (**I**) ROS generation, and (**J**) ATP levels in osteoclasts treated with vector-E*G*FP or *SIRT3*-EGFP in RAW264.7 cells. * adjusted *p* < 0.05, ** adjusted *p* < 0.01, *** adjusted *p* < 0.001, ns, not significant; error bars = SD; data are presented as mean values ± SD.

To study the effect of *SIRT3* on mitochondrial function, mitochondrial status and ROS levels were assessed after *SIRT3* knockdown and overexpression. After *SIRT3* knockdown, the mitochondrial membrane potential increased slightly during osteoclast differentiation, and the inhibitory effect of SZQ-4 on the mitochondrial membrane potential of osteoclasts was weakened (Figure 6A,C). Similarly, *SIRT3* knockdown increased mitochondrial ROS levels during osteoclast differentiation, and the effect of SZQ-4 on clearing mitochondrial ROS in osteoclasts was reduced (Figure 6B,D).

After *SIRT3* knockdown, ATP levels significantly increased during osteoclast differentiation, and SZQ-4 reduced the inhibition of mitochondrial ATP production (Figure 6E). When *SIRT3* was overexpressed in RANKL-induced osteoclasts, mitochondrial membrane potential, mitochondrial ROS levels, and ATP levels significantly decreased, but SZQ-4’s effect on *SIRT3* was minimal (Figure 6F–J). In conclusion, *SIRT3* knockdown reduced the effect of SZQ-4 on mitochondrial function in osteoclasts. When *SIRT3* was overexpressed, SZQ-4 had no significant impact on the mitochondrial function of osteoclasts, possibly because the mitochondria reached a stable state due to the action of SZQ-4. These results suggest that SZQ-4 regulates mitochondrial function via *SIRT3*.

### 3.7. SZQ-4 Treatment Prevented OVX-Induced Bone Loss, Reduced Osteoclast Numbers, ROS Production, and SIRT3 Expression In Vivo

**Figure 7:**
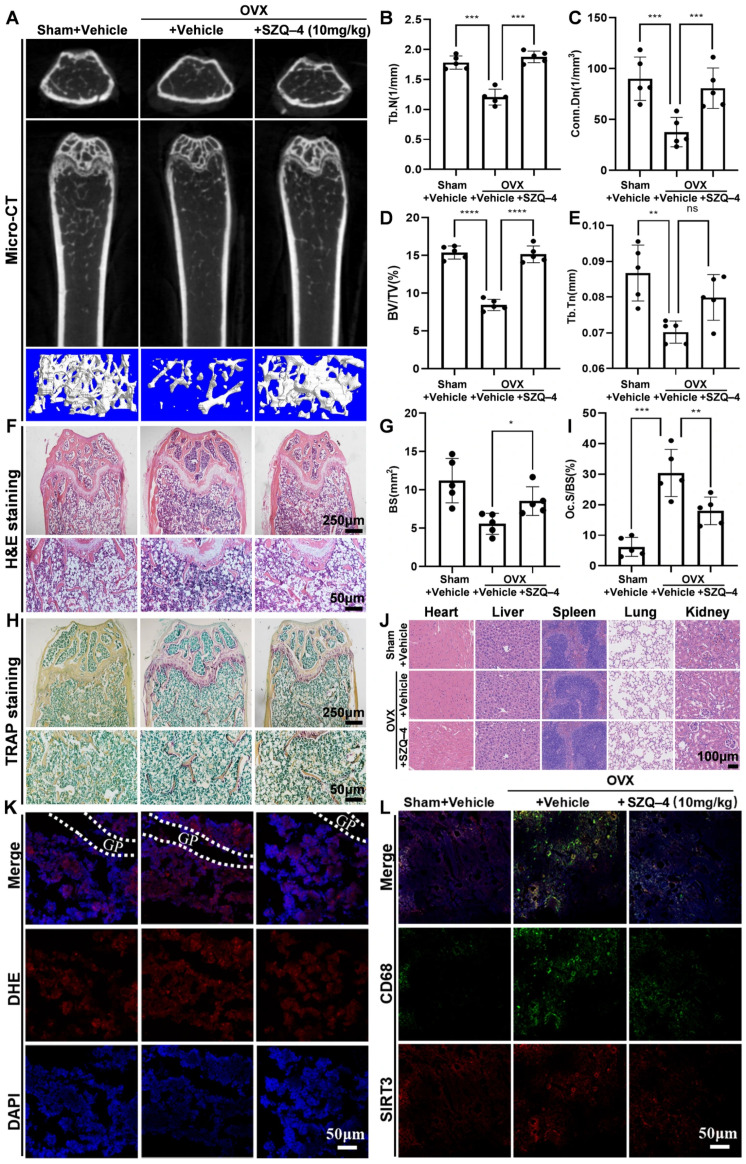
SZQ-4 treatment prevents OVX-induced bone loss and reduces osteoclasts in vivo. (**A**) Representative 2D and 3D micro-CT scanning images of femur tissue indicate that SZQ-4 intervention prevents bone loss. (**B**–**E**) Quantitative analysis of bone microstructure parameters, including BV/TV, Tb.N, Conn.Dn, and Tb.Th. (**F**,**G**) Representative H&E staining images of femur tissue sections and corresponding statistical analysis (*n* = 5 mice per group). (**H**,**I**) Representative TRAP staining images of femur tissue sections and corresponding statistical analysis. (**J**) major organ section images stained with H&E of heart, liver, spleen, lung, and kidney. (**K**) Representative DHE staining images of frozen femur sections (GP: growth plate). (**L**) Representative immunofluorescence staining images of femur tissue sections. * adjusted *p* < 0.05, ** adjusted *p* < 0.01, *** adjusted *p* < 0.001, **** adjusted *p* < 0.0001, ns, not significant; error bars = SD; data are presented as mean values ± SD.

These results showed that SZQ-4 inhibits osteoclast formation and function in vitro. To assess SZQ-4’s potential as a prophylactic agent, a mouse model of osteoporosis induced by ovarian removal was used. Mice underwent OVX or sham surgery and were administered SZQ-4 (10 mg/kg) by oral gavage every 2 days or with vehicle for 8 weeks postoperatively. Micro-CT confirmed that SZQ-4 prevented extensive bone loss in mice after OVX femoral surgery (Figure 7A).

Quantitative analysis of bone parameters revealed that SZQ-4 treatment significantly increased BV/TV, Tb.N, Tb.Th, and Conn.Dn compared to untreated OVX mice (Figure 7B–E). Histological examination consistently showed that SZQ-4 reduced OVX-induced bone loss (Figure 7F). Quantification of H&E staining showed that bone volume and surface area were well maintained in the SZQ-4-treated group compared to OVX mice untreated with SZQ-4 (Figure 7G). Histological staining with TRAP was performed on femoral sections to examine osteoclast activity in vivo. Figure 7H,I demonstrate that OVX resulted in an increased area of TRAP-positive osteoclasts, whereas SZQ-4 treatment prevented this increase in osteoclast area in vivo. This was supported by the quantification of osteoclast parameters, which demonstrated a reduction in the surface area of osteoclasts on the bone surface of SZQ-4-treated mice.

Figure 7J shows that SZQ-4 caused no apparent inflammatory cell infiltration or pathological changes in the organ tissues, exhibiting good biosafety in vivo.

Figure 7K shows that the ROS content indicated by red fluorescence was higher in the bone tissue of the control mice compared to the sham-operated group. SZQ-4 treatment reduced ROS content in the bone tissue. Immunofluorescence staining revealed that when green fluorescence, representing CD68, overlapped with red fluorescence, representing *SIRT3*, *SIRT3* was localized in osteoclasts. These results showed that *SIRT3* expression in the bone tissue of mice in the control group was higher than in the SZQ-4 treatment group (Figure 7L). These results demonstrated that SZQ-4 treatment reduced ROS production and *SIRT3* expression in OVX mice, thereby reducing osteoclast differentiation (Figure S2).

## 4. Discussion

During RANKL-induced differentiation of BMDMs into osteoclasts, both the number and size of mitochondria increase, with mature osteoclasts containing numerous mitochondria [39]. Notably, mitochondria consume 85–90% of cellular oxygen, producing ATP and over 90% of cellular ROS [36]. High ROS levels stimulate osteoclasts to generate a series of signal transducers and promote osteoclast differentiation [40]. Mitochondrial acetylation is a key regulatory mechanism of mitochondrial function, primarily controlled by the NAD-dependent deacetylase *SIRT3* [19,41,42]. As a member of the SIRT family, *SIRT3* is located in the mitochondrial inner membrane and regulates ATP production, oxidative stress, energy metabolism, and mitochondrial homeostasis. Oxidative phosphorylation is the primary source of ATP in aerobic organisms and is crucial for energy-demanding cells such as osteoclasts [43]. During osteoclastogenesis, ROS are produced in the electron transport chain of the inner mitochondrial membrane. RANKL induces *SIRT3* expression to meet the demand for controlling oxidative stress, as mitochondrial biogenesis increases during osteoclastogenesis. In addition, *SIRT3* deacetylates SOD2, thereby enhancing SOD2 activity and reducing ROS levels in osteoclasts, thereby inhibiting osteoclast differentiation [44]. By mitigating intracellular oxidative stress, *SIRT3* also improves mitochondrial function [45].

In this study, transcriptome sequencing analysis revealed that *SIRT3* significantly impacted SZQ-4′s inhibition of osteoclast differentiation. To further clarify *SIRT3*′s mechanism of action, we knocked down and overexpressed *SIRT3* in RAW264.7 and measured mitochondrial function and ROS levels. These results demonstrate that *SIRT3* and ROS jointly regulate mitochondrial homeostasis and are crucial for SZQ-4′s inhibition of osteoclast differentiation. However, we acknowledge that our current data do not distinguish whether SZQ-4 directly modulates *SIRT3* or indirectly affects its expression through ROS reduction. Future studies employing *SIRT3* activity assays and upstream regulator analysis will be necessary to clarify the precise molecular interaction between SZQ-4 and *SIRT3*. In addition, we conducted a series of in vitro experiments to demonstrate that SZQ-4 inhibited osteoclastogenesis by reducing intracellular ROS levels, thereby regulating *SIRT3* expression and maintaining mitochondrial homeostasis. Using RNA-seq, WB, and qRT-PCR techniques, our study revealed that SZQ-4 inhibits osteoclast differentiation by alleviating ROS and regulating *SIRT3*. Animal studies using an OVX-induced osteoporosis mouse model demonstrated that SZQ-4 (10 mg/kg/d) improved bone mass, microstructure, and strength by inhibiting bone resorption. While micro-CT provides detailed information on bone microarchitecture and has been shown to correlate with mechanical properties [46,47,48], structural preservation alone does not fully reflect functional bone competence. The absence of direct biomechanical measurements limits conclusions regarding actual load-bearing capacity. Accordingly, future investigations integrating biomechanical testing are warranted to better define the functional and therapeutic efficacy of SZQ-4.

In addition, as a flavonoid and maleimide compound, SZQ-4 not only clears free radicals, prevents other oxidants, chelates metal ions, and improves the activity and expression of antioxidant enzymes but also has antitumor, antibacterial, and other biological activities [49]. Given its structure, SZQ-4 is hypothesized to maintain bone homeostasis by inhibiting osteoclast differentiation, reducing intracellular ROS levels, and contributing to bone remodeling and homeostasis. Additionally, SZQ-4′s significant anti-inflammatory effects suggest that it may also address inflammation-driven osteoclast overactivation, which can lead to osteoporosis. 4-Methyltyrosol (4-MC), a bioactive phenolic compound, acts as an agonist of neurotrophic factors. It binds to *Keap1* (Gly367/Ile559/Val606), activating *Nrf2* to reduce ROS, suppress *NF-κB* phosphorylation, and mitigate osteoporosis in OVX mice, while inhibiting osteoclast differentiation in vitro [50]. While both SZQ-4 and 4-MC function as ROS-scavenging agents, our data indicate that SZQ-4 operates through a distinct, earlier regulatory node centered on *SIRT3*-mediated mitochondrial function and ROS metabolism. Whether the ROS-scavenging compound SZQ-4 similarly modulates bone loss via the ROS/*Keap1*/*Nrf2* axis warrants further investigation. However, these findings are based on in vitro experiments, and further validation using *SIRT3*-KO or *SIRT3*-flox mice is needed to confirm the in vivo interaction between *SIRT3* and ROS. Future studies should comprehensively investigate the effects of SZQ-4 on other bone cell populations—including osteoblasts, osteocytes, and their progenitors—to fully delineate its broader role in bone homeostasis and elucidate how its modulation of antioxidant pathways influences the coordinated cellular activities underlying bone remodeling.

## 5. Conclusions

In summary, this study evaluated the potential of SZQ-4, a novel synthetic flavone–maleimide hybrid, as a regulator of bone resorption for the treatment of postmenopausal osteoporosis. SZQ-4 primarily inhibited osteoclast formation and differentiation by reducing ROS production, altering *SIRT3* expression, and stabilizing mitochondrial function, thereby preventing extensive bone loss in vivo and restoring bone resorption in an ovariectomy-induced bone loss mouse model. SZQ-4 also demonstrated oral bioactivity and favorable biosafety for chronic use, making it a promising candidate for treating osteoporosis, particularly in postmenopausal patients. Future studies should investigate the effects of SZQ-4 on other bone cell types.

## Data Availability

The original contributions presented in this study are included in the article/Supplementary Material. The original RNA sequencing data generated in this study are openly available in the NCBI Sequence Read Archive (SRA) under BioProject accession number PRJNA1428510. Further inquiries can be directed to the corresponding authors.

## Supplementary Materials

The following supporting information can be downloaded at: https://www.mdpi.com/article/10.3390/antiox15040426/s1. Figure S1: SZQ-4 inhibited podosome belt formation and inhibits osteoclast resorptive function; Figure S2: SZQ-4 treatment reduces ROS production and *SIRT3* expression in OVX mice; Table S1: Key resources; Table S2: Primers used for quantitative PCR; Table S3: *Sirt3* Plasmid Information.

## Abbreviations

The following abbreviations are used in this manuscript:

ABTS	2,2′-Azinobis-(3-ethylbenzothiazoline-6-sulfonic acid)
ATP	Adenosine triphosphate
BMDMs	Bone marrow–derived macrophages
BV/TV	Bone volume per tissue volume
Conn.Dn	Connectivity density
CTSK	Cathepsin K
DC-STAMP	Dendritic cell–specific transmembrane protein
DHE	Dihydroethidium
DMSO	Dimethyl sulfoxide
DPPH	2,2-Diphenyl-1-picrylhydrazyl
FCCP	Carbonyl cyanide-4-(trifluoromethoxy)phenylhydrazone
FBS	Fetal bovine serum
GO	Gene Ontology
H&E	Hematoxylin and eosin
IF	Immunofluorescence
M-CSF	Macrophage colony-stimulating factor
MMP	Mitochondrial membrane potential
mtROS	Mitochondrial reactive oxygen species
NFATc1	Nuclear factor of activated T cells 1
OCR	Oxygen consumption rate
OVX	Ovariectomy/ovariectomized
PGC-1β	Peroxisome proliferator-activated receptor gamma coactivator 1 beta
PMO	Postmenopausal osteoporosis
qPCR	Quantitative polymerase chain reaction
qRT-PCR	Quantitative real-time polymerase chain reaction
RANKL	Receptor activator of nuclear factor kappa-B ligand
ROS	Reactive oxygen species
siRNA	Small interfering RNA
SIRT3	Sirtuin 3
SOD2	Superoxide dismutase 2
SZQ-4	Synthetic flavonoid compound SZQ-4
Tb.N	Trabecular number
Tb.Th	Trabecular thickness
TRAP	Tartrate-resistant acid phosphatase
