# Supplementary material for: Synthetic Novel Flavonoids SZQ-4 Suppress Osteoclastogenesis and Ameliorate Osteoporosis via Inhibiting Reactive Oxygen Species and Regulating SIRT3
Source: Antioxidants (Basel). 2026 Mar 28;15(4):426. doi: 10.3390/antiox15040426 (PMC13113758; doi:10.3390/antiox15040426)
Supplement: Supplementary file 1 [file antioxidants-15-00426-s001.zip › Supplementary Figure.pdf]

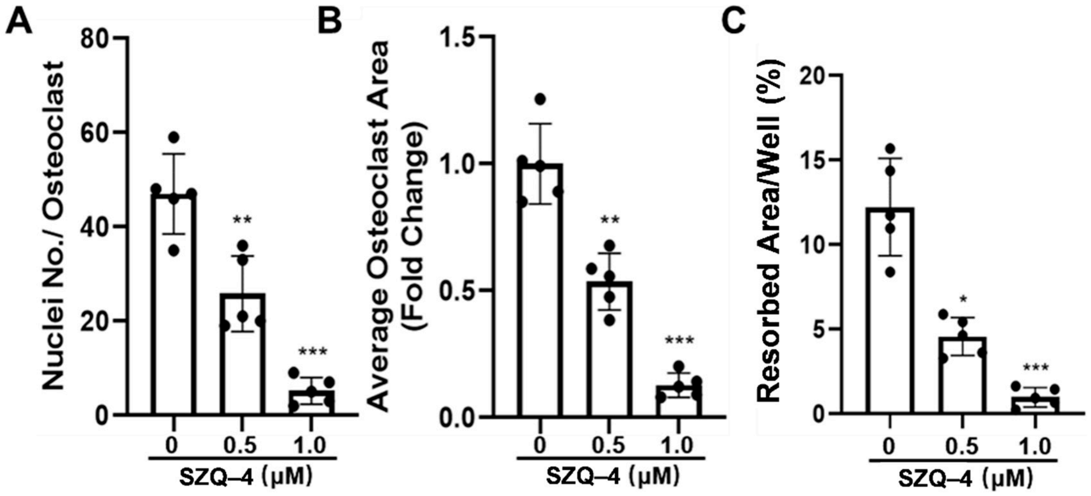

Supplementary Figure S1 SZQ-4 inhibited podosome belt formation and inhibits osteoclast resorptive function. (A) Quantification of the nuclei number per osteoclast. (B) Quantification of the average osteoclast area. (C) Quantification of hydroxyapatite resorption area per well (*n* = 5 independent samples). *adjusted *P* < 0.05, ***adjusted *P* < 0.001; error bars = SD; data are presented as mean values ± SD.

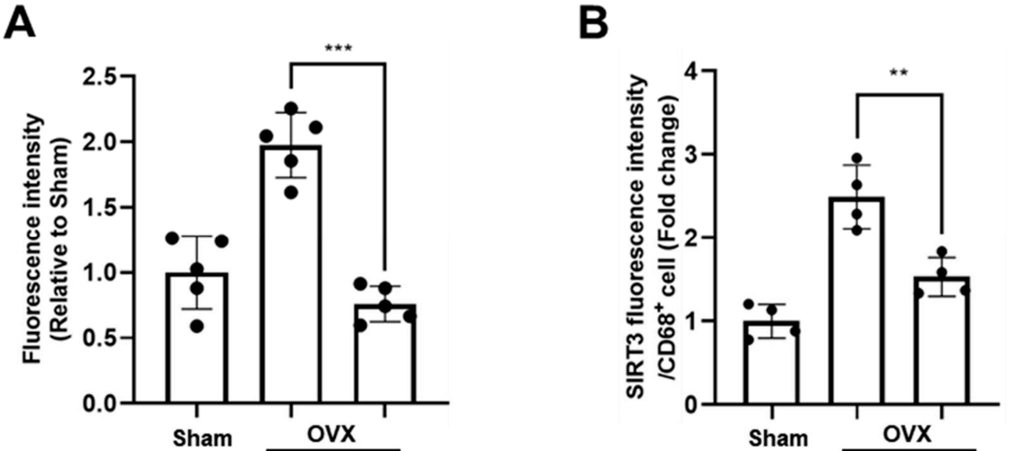

Supplementary Figure S2 SZQ-4 treatment reduces ROS production and SIRT3 expression in OVX mice. (A) Analysis of reactive oxygen levels in femur sections. (B) Analysis of the expression level of SIRT3 in osteoclasts. **adjusted P < 0.01, ***adjusted P < 0.001; error bars = SD; data are presented as mean values ± SD.
