# Supplementary material for: Synthetic Novel Flavonoids SZQ-4 Suppress Osteoclastogenesis and Ameliorate Osteoporosis via Inhibiting Reactive Oxygen Species and Regulating SIRT3
Source: Antioxidants (Basel). 2026 Mar 28;15(4):426. doi: 10.3390/antiox15040426 (PMC13113758; doi:10.3390/antiox15040426)
Supplement: Supplementary file 1 [file antioxidants-15-00426-s001.zip › Supplementary Table.pdf]

Supplementary Table S1 Key resources

Reagent or resources	Source	Identifier
Antibodies		
SIRT3	Cell Signaling Technology	5490
NFATC1	Cell Signaling Technology	8032
β-actin	Cell Signaling Technology	4967
Chemicals and recombinant proteins		
Alpha-modified minimal essential medium (α-MEM)	Gibco	6125188
Opti-MEM	Gibco	31985070
Rhodamine-phalloidin	Invitrogen	R415
Fetal bovine serum	Sigma	102801691
Dimethyl sulfoxide (DMSO)	Sigma	D5879-1L
Trizol reagent	Sigma	T9424
4,6-diamidino-2-phenylindole (DAPI)	Beyotime	C1002
2',7'-dichlorodihydrofluorescein diacetate (H2DCFDA)	Invitrogen	C2938
Recombinant mouse RANKL	R&D Systems	462-TEC
Recombinant mouse M-CSF	R&D Systems	416-ML-050/CF
Dihydroethidium	Apex Bio	104821-25-2
Penicillin-Streptomycin	Solarbio	P1400
0.25% Trypsin-EDTA	Gibco	25200072
PBS	Servicebio	G4202
Skim milk	BioFroxx	1172
EDTA Na_2	Solarbio	E8030
PAGE Pre-Solution	Solarbio	A1010
Glycine	Solarbio	G8200
Trizma® base	Sigma	V900483-5KG
DEPC-H_2O	Generay biotech	D1007
Sodium dodecylsulfate (SDS)	Solarbio	S8012
Sodium Chloride (NaCl)	Solarbio	S8210
Sodium hydroxide (NaOH)	Mackin	S817979
Precision Plus Protein Dual Color Standards	Bio-Rad	1610374
HRP-labeled Goat Anti-Rabbit IgG	Beyotime	A0208
HRP-labeled Goat Anti-Mouse IgG	Beyotime	A0216
AF647-labeled Goat Anti-Rabbit IgG	Beyotime	A0468
AF488-labeled Goat Anti-Mouse IgG	Beyotime	A0428
Critical commercial assays		
Image-iT™ TMRM assay kit	Invitrogen	I34361
MitoSOX Red assay kit	Invitrogen	M36008
MitoTracker™ Dyes for Mitochondria Labeling	Invitrogen	M7514

leukocyte acid phosphatase staining kit	Sigma	387A-1KT
Lipofectamine™ 3000	Invitrogen	L3000075
MTS assay kit	Sigma	475989
CellTiter-Glo® Luminescent Cell Viability Assay	Promega	G7572
HiScript Q RT SuperMix for qPCR	Vazyme	R123-01
ChamQ Universal SYBR qPCR Master Mix	Vazyme	Q711
ABTS Free Radical Scavenging Capacity Assay Kit	Solarbio	BC4770
DPPH Free Radical Scavenging Capacity Assay Kit	Solarbio	BC4750

Supplementary Table S2. Primers used for quantitative PCR

Gene	Gene ID	Primer Forward (5′ to 3′)	Primer Reverse (5′ to 3′)
c-Fos	14281	CCGAAGGGAACGGAATAAGATGG	CCTCTGGGAAGCCAAGGTCAT
MMP9	17395	AGTGGGACCATCATAACATCACATACT	TGCTCCGCGACACCAAACT
CTSK	13038	GTATAACGCCACGGCAAAGG	CATAGCCCACCACCAACACTG
NFATc1	18018	ACCTGTGCAAGCCAAATTCCC	TCAAAGTCGTCCGTGGGTTCT
TRAP	11433	ACGATGCCAGCGACAAGAGG	TGAAGCGCAAACGGTAGTAAGG
DC-STAMP	75766	TCCAGCATTTGGGAGTCTGTTG	ATCAAAGCGTTCCTACCTTCACG
Sirt3	64384	GGCACTACAGGCCCAATGT	CTCTCAAGCCCGTCGATGT
β-actin	11461	GCTGTCCCTGTATGCCTCTG	TTGATGTCACGCACGATTTCCC

Supplementary Table S3. Sirt3 Plasmid Information

Sirt3 Plasmid Information Sheet

Gene ID	64384
Gene Symbol	*Sirt3*
Full Gene Name	sirtuin 3
Aliases	Sir2l3; 2310003L23Rik
Transcript Accession No.	NM_001177804
CDS Length	1005 bp
Vector Tag	None
Cloning Sites	EcoRI/BstBI
Sequencing Primers	Forward (CAG): 5′-CTCTGCTAACCATGTTCATG-3′
	Reverse (GFP): 5′-CCGTTCAGGGTGCCGGTG-3′
Selectable Markers	Prokaryotic: Ampicillin (100 μg/mL); Eukaryotic: Puromycin
Gene Sequence	No mutations

> *Sirt3* (without stop codon)
ATGGCGCTTGACCCTCTAGGCGCCGTCGTCCTGCAGAGCATCATGGCGCTAAGCGGTC
GACTGGCATTGGCCGCGCTCAGACTGTGGGGTCCGGGAGGTGGGAGAAGGCCCATAT
CCCTCTGTGTGGGAGCCTCAGGCGGCTTTGGAGGTGGAGGAAGCAGTGAGAAGAAGT
TTTCTCTGCAGGATGTAGCTGAGCTGCTTCGGACCAGAGCCTGCAGTAGGGTGGTGGT
CATGGTGGGGGCCGGCATCAGCACACCCAGTGGCATCCCGGACTTCAGATCCCCAGGG
AGCGGCCTCTACAGCAACCTTCAGCAGTATGACATCCCGTACCCTGAAGCCATCTTTGA
ACTTGGCTTTTTCTTTCACAACCCCAAGCCCTTTTTCATGTTGGCCAAGGAGCTGTACC
CTGGGCACTACAGGCCCAATGTCACTCACTACTTCCTGAGGCTCCTCCACGACAAGGA
GCTGCTTCTGCGGCTCTATACACAGAACATCGACGGGCTTGAGAGAGCATCTGGGATC
CCTGCCTCAAAGCTGGTTGAAGCCCACGGGACCTTTGTAACAGCTACATGCACGGTCT
GTCGAAGGTCCTTCCCAGGGGAAGACATATGGGCTGATGTGATGGCGGACAGGGTGCC
CCGCTGCCCTGTCTGTACTGGCGTTGTGAAACCCGACATTGTGTTCTTTGGGGAGCAG
CTGCCTGCAAGGTTCCTACTCCATATGGCTGACTTCGCTTTGGCAGATCTGCTACTCATT
CTTGGGACCTCCCTGGAGGTGGAGCCTTTTGCCAGCTTGTCTGAAGCAGTACAGAAAT
CAGTGCCCCGACTGCTCATCAATCGAGACTTGGTGGGGCCGTTCGTTCTGAGTCCTCG
AAGGAAAGATGTGGTCCAGCTAGGGGATGTAGTTCATGGTGTGGAAAGGCTGGTGGA
CCTCCTGGGGTGGACACAAGAACTGCTGGATCTTATGCAGCGGGAACGTGGCAAGCT
GGATGGACAGGACAGA

Plasmid map

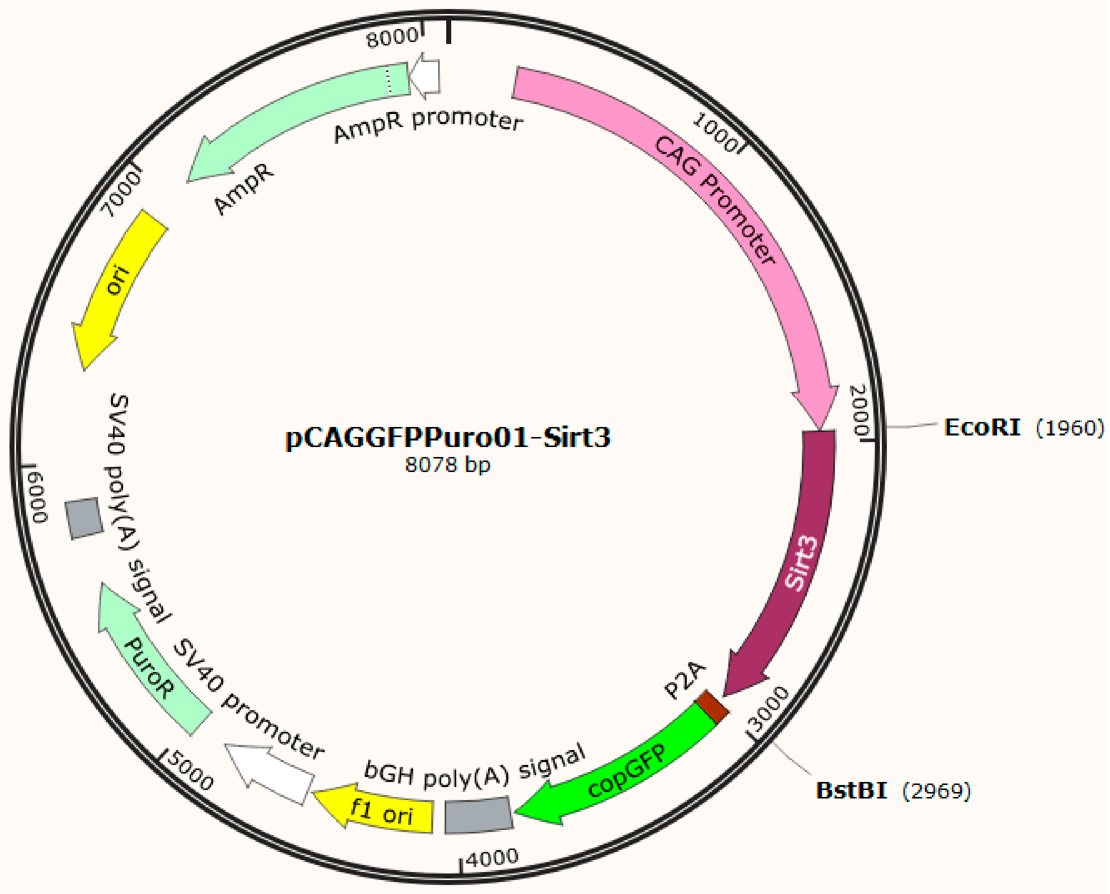

pCAGGFPPuro01-Sirt3
8078 bp
CAG Promoter
Sirt3
P2A
copGFP
bGH poly(A) signal
f1 ori
SV40 promoter
PuroR
SV40 poly(A) signal
ori
AmpR
AmpR promoter
EcoRI (1960)
BstBI (2969)
1000
2000
3000
4000
5000
6000
7000
8000
